# Maternal Emergency Department Use Before Pregnancy and Infant Emergency Department Use After Birth

**DOI:** 10.1001/jamanetworkopen.2023.2931

**Published:** 2023-03-13

**Authors:** Catherine E. Varner, Alison L. Park, Joel G. Ray

**Affiliations:** 1 Schwartz/Reisman Emergency Medicine Institute, Toronto, Ontario, Canada; 2 Department of Emergency Medicine, Mount Sinai Hospital, Toronto, Ontario, Canada; 3 Department of Family and Community Medicine, University of Toronto, Toronto, Ontario, Canada; 4 ICES, Toronto, Ontario, Canada; 5 Keenan Research Centre, Li Ka Shing Knowledge Institute, St. Michael’s Hospital, Toronto, Ontario, Canada; 6 Departments of Medicine, and Obstetrics and Gynaecology, St Michael’s Hospital, Toronto, Ontario, Canada

## Abstract

**Importance:**

Maternal emergency department (ED) use before or during pregnancy is associated with worse obstetrical outcomes, for reasons including preexisting medical conditions and challenges in accessing health care. It is not known whether maternal prepregnancy ED use is associated with higher use of the ED by their infant.

**Objective:**

To study the association between maternal prepregnancy ED use and risk of infant ED use in the first year of life.

**Design, Setting, and Participants:**

This population-based cohort study included all singleton livebirths in all of Ontario, Canada, from June 2003 to January 2020.

**Exposures:**

Any maternal ED encounter within 90 days preceding the start of the index pregnancy.

**Main Outcomes and Measures:**

Any infant ED visit up to 365 days after the index birth hospitalization discharge date. Relative risks (RR) and absolute risk differences (ARD) were adjusted for maternal age, income, rural residence, immigrant status, parity, having a primary care clinician, and number of prepregnancy comorbidities.

**Results:**

There were 2 088 111 singleton livebirths; the mean (SD) maternal age was 29.5 (5.4) years, 208 356 (10.0%) were rural dwelling, and 487 773 (23.4%) had 3 or more comorbidities. Among singleton livebirths, 206 539 mothers (9.9%) had an ED visit within 90 days before the index pregnancy. ED use in the first year of life was higher among infants whose mother had visited the ED before pregnancy (570 per 1000) vs those whose mother had not (388 per 1000) (RR, 1.19 [95% CI, 1.18-1.20]; ARD, 91.1 per 1000 [95% CI, 88.6-93.6 per 1000]). Compared with mothers without a prepregnancy ED visit, the RR of infant ED use in the first year was 1.19 (95% CI, 1.18-1.20) if its mother had 1 prepregnancy ED visit, 1.18 (95% CI, 1.17-1.20) following 2 visits, and 1.22 (95% CI, 1.20-1.23) after at least 3 maternal visits. A low-acuity maternal prepregnancy ED visit was associated with an adjusted odds ratio (aOR) of 5.52 (95% CI, 5.16-5.90) for a low-acuity infant ED visit, which was numerically higher than the pairing of a high-acuity ED use between mother and infant (aOR, 1.43, 95% CI, 1.38-1.49).

**Conclusions and Relevance:**

In this cohort study of singleton livebirths, prepregnancy maternal ED use was associated with a higher rate of ED use by the infant in the first year of life, especially for low-acuity ED use. This study’s results may suggest a useful trigger for health system interventions aimed at reducing some ED use in infancy.

## Key Points

**Question:**

What is the association between maternal prepregnancy emergency department (ED) use and infant ED use in the first year of life?

**Findings:**

This population-based cohort study of 2 088 111 singleton livebirths was completed within a universal health care system. An ED visit within 90 days before the start of pregnancy was associated with a higher risk of an infant ED visit(s), and low-acuity maternal ED visits were most highly associated with low-acuity infant ED visits.

**Meaning:**

This study’s results suggest that prepregnancy ED use forecasts a higher rate of ED use in infants, and it potentially offers a useful trigger for health system interventions to decrease acute care utilization for low acuity indications in infancy.

## Teaser

This cohort study examines whether there is an association between maternal prepregnancy emergency department (ED) use and risk of infant ED use in the first year of life.

## Introduction

Emergency departments (EDs) are under enormous strain, with rising use among persons of all ages, especially children and infants.^1,2,3,4,5^ ED use not only reflects a patient’s current illness or degree of medical complexity, but is an indicator of their access to primary care and availability of specialist and community-based supports.^6,7,8,9,10^ Among all children, infants less than 1 year of age have the highest rates of ED use,^4,7^ with recurrent ED use a sign of uncoordinated health care access and economic disparity.^11^

ED use in pregnancy is common and is also reflective of poorer access to antenatal care,^12^ with worse ensuing maternal and perinatal outcomes.^13^ Even prepregnancy ED access is associated with a higher risk of severe maternal morbidity, severe neonatal morbidity, stillbirth, and neonatal death, especially as the number of prepregnancy ED visits increases.^14^

Prior research observed a higher rate of ED encounters among infants whose mother was affected by a mental health problem or greater medical comorbidity, younger maternal age, and ED visits during pregnancy.^11,15^ A major limitation of these studies is that they were restricted to commercially insured or low-income patients in the US, in contrast to regions with universal access to physician and hospital care, such as in Canada. Furthermore, to our knowledge, completed research did not consider prepregnancy maternal ED use, which would be exclusive of any new-onset maternal or fetal condition arising in pregnancy or soon after birth. Such a novel approach might better test the hypothesis that a woman’s reduced access to sufficient primary care, and hence, greater ED use before conception, translates into greater ED use by their newborn infant. Accordingly, the current Canadian study examined the association between maternal prepregnancy ED use and infant ED use in the first year of life.

## Methods

This retrospective population-based cohort was completed within all of Ontario, Canada. Ontario is Canada’s most populous province that has universal health care coverage and standardized collection of all outpatient, ED, and inpatient hospital care services.

Data sets were linked using unique encoded identifiers and analyzed at ICES. Use of data in this project was authorized under section 45 of Ontario’s Personal Health Information Protection Act, which does not require review by a research ethics board nor informed consent. Reporting of this cohort study’s findings was consistent with the Strengthening the Reporting of Observational Studies in Epidemiology (STROBE) reporting guideline.

### Study Population

Included were all mothers aged 10 to 55 years with a singleton hospital livebirth in Ontario between June 1, 2003, and January 31, 2020. Excluded were those without a valid Ontario Health Insurance Plan (OHIP) number, non-Ontario residents, births before 20 weeks’ or after 42 weeks’ gestation, and any liveborn infant who was not alive at the index hospital birth discharge date (eFigure in Supplement 1).

### Data Sources

This study used validated health administrative databases for the entire province of Ontario, including the Canadian Institute for Health Information Discharge Abstract Database (DAD), the Same Day Surgery Database and National Ambulatory Care Reporting System (NACRS) database, the OHIP claims database, and the ICES MOMBABY database, which links maternal hospital deliveries to newborn hospital births^16,17,18,19^ (eTable 1 in Supplement 1). Each mother and newborn has their own unique encoded identifier number. Residential income quintile and rural residence were based on Statistics Canada census data, using the 6-digit maternal postal code.^20^ Date of conception is estimated using the clinical gestational weeks at delivery on the hospital birth record, the latter of which is based on accurate pregnancy dating by a first- or second-trimester ultrasound for greater than 95% of births in Ontario.^21^

### Exposures and Outcomes

The main exposure was any maternal prepregnancy ED encounter within 90 days before conception (time zero). Time zero was the estimated conception date of the index pregnancy minus 2 weeks earlier–equivalent to the first day of a person’s last menstrual period–to account for any potential inaccuracy in pregnancy dating, and therefore, to ensure that a person was not yet pregnant.

An ED is formally recognized as a hospital facility that serves unscheduled patients, whose condition may require immediate care; it must be staffed by a physician at all times. All ED visits were identified in the NACRS database, defined as an ED encounter between a patient seeking care and a physician, or a physician assistant or nurse practitioner working under a physician’s supervision (eTable 1 in Supplement 1). An ED encounter that resulted in an ED discharge or in a hospital admission was included.

The primary study outcome was any infant ED visit within 365 days after the index birth hospitalization discharge date. Secondary outcomes included infant death within 365 days after the index birth hospitalization discharge date, as well as infant readmission to hospital within 365 days after the index birth hospitalization discharge date.

### Statistical Analysis

Baseline variables were contrasted using standardized differences, comparing mothers who did vs mothers who did not have an ED visit within 90 days before the index pregnancy. For a given variable, a standardized difference greater than 0.10 denoted an important difference between groups.

Modified Poisson regression with a robust error variance^22^ was used to generate relative risks (RRs), absolute risk differences, and 95% CI for the association between a mother having any vs no ED visit within 90 days before pregnancy, and each respective study outcome. To account for potentially more than 1 birth per mother during the study period, generalized estimating equations with an exchangeable correlation structure accounted for correlated errors. RR were adjusted for maternal age, area-level income quintile, rural residence, immigrant status, and parity—each at the start of the index pregnancy; having a primary care clinician within 365 days before the start of the index pregnancy; as well as the number of comorbidities within 120 days before the start of the index pregnancy. Comorbidities were expressed as the total number of Johns Hopkins Adjusted Clinical Groups (ACG) System Aggregated Diagnosis Groups ([ADG]: ≤2, 3-4, 5-6, and 7-32). This analysis was further stratified by newborn sex, preterm birth less than 37 weeks’ gestation, and the presence of severe neonatal morbidity during the index birth hospitalization, as described in eTable 1 in Supplement 1. For this analysis, we also calculated an E-value for the adjusted association (ie, aRR) between maternal prepregnancy ED use and infant ED use.^23^ A larger E-value suggests that a stronger unmeasured confounder is necessary to refute the observed association, and a value of 1 indicating that no unmeasured confounding is needed to explain away the observed association.

In a dose-response analysis, the risk of any infant ED visit within 365 days after the birth discharge was evaluated in comparison with the number of maternal prepregnancy ED encounters, categorized as 0 (referent), 1, 2, or at least 3 ED visits, and otherwise modeled as aforementioned. Next, using multinomial logistic regression, the odds of an infant having a nonurgent ED visit within 365 days after the birth discharge date was calculated in comparison with its mother having a nonurgent ED visit within 90 days before the pregnancy. If more than 1 ED visit occurred, the mother’s latest visit was used within 90 days before conception, whereas the infant’s first ED visit was used within 1 year after birth discharge. The Canadian Triage and Acuity System (CTAS) is a 5-level triage tool that is used to prioritize the order in which ED patients should be seen and is used at the point of care in all of Ontario EDs. A higher CTAS score (CTAS 4 or 5) indicates a less urgent visit.^24^ Odds ratios (ORs) were adjusted for the same variables as previously mentioned.

The RR of any infant ED visit in the first year of life was also evaluated in comparison with the cumulative number of minutes its mother had spent in the ED before conception, the latter expressed in deciles, and each relative to those with no ED visit (referent). To be clear, this analysis permitted a mother to have more than 1 ED visit before pregnancy, as the exposure was the total ED length of stay across the 90-day period before the index pregnancy. RRs were adjusted for the same covariates.

In NACRS, each patient’s’s ED visit is assigned a “Main Problem” (ie, main diagnosis), grouped into one of the existing chapters of the *International Statistical Classification of Diseases and Related Health Problems, Tenth Revision, Canada (ICD-10-CA)*.^25^ Accordingly, the RR for any infant ED visit within 365 days after birth discharge was assessed in comparison with the Main Problem group at its mother’s most recent ED visit within the 90-day prepregnancy period, relative to those with no ED visit (referent).

Analyses were conducted using SAS statistical software version 9.4 for Unix (SAS Institute) and the Johns Hopkins ACG System Version 10. Statistical significance was set at *P* < .05, and all tests were 2-tailed. All cell sizes under 6 were suppressed to prevent patient reidentification. As all births in the study period were included, and no sample size calculation was performed.

## Results

There were 2 221 223 eligible pregnancies identified, of which 133 112 (6.0%) were excluded, owing primarily to multifetal births, invalid OHIP number, extreme maternal age, or non-Ontario residency (eFigure in Supplement 1). Among all 2 088 111 singleton livebirths, 208 356 (10.0%) were rural dwelling and 487 773 (23.4%) had 3 or more comorbidities; the mean (SD) maternal age was 29.5 (5.4) years; 206 539 mothers (9.9%) had an ED visit within 90 days before the index pregnancy, with the most recent ED visit occurring at a median (IQR) of 43 (22-66) days preceding time zero. In contrast to those who did not visit the ED prepregnancy, mothers who used the ED were more likely to be younger, reside in a lower income or rural area, to be a nonimmigrant, and to have a greater number of ADGs (Table 1). Of mothers without a prepregnancy ED visit, 1 732 028 (92.1%) had a primary care clinician, in contrast to 199 884 (96.8%) of those who had an ED visit 90 days before conception.

**Table 1.** Characteristics of All Mothers and Their Singleton Newborns From April 2003 to January 2020, Categorized by Maternal ED Visit Within 90 Days Before the Index Pregnancy Conception Date

Maternal characteristic	Mother with any ED visit within 90 d before pregnancy, No. (%)		Absolute standardized difference^a^
	ED visit (n = 206 539)	No ED visit (n = 1 881 572)	
At start of the index pregnancy			
Age, y			
Mean (SD)	27.7 (5.9)	29.7 (5.3)	0.38
10-19	19 717 (9.5)	65 731 (3.5)	0.25
20-24	44 649 (21.6)	243 090 (12.9)	0.22
25-29	61 844 (29.9)	569 823 (30.3)	0.01
30-34	53 605 (26.0)	653 473 (34.7)	0.19
35-39	22 886 (11.1)	296 437 (15.8)	0.14
40-44	3691 (1.8)	50 487 (2.7)	0.06
45-55	147 (0.1)	2531 (0.1)	0.02
Residential income quintile			
1 (lowest)	55 907 (27.1)	419 368 (22.3)	0.11
2	43 868 (21.2)	379 674 (20.2)	0.03
3	40 273 (19.5)	383 778 (20.4)	0.02
4	37 490 (18.2)	384 555 (20.4)	0.06
5 (highest)	27 820 (13.5)	307 382 (16.3)	0.08
Missing	1181 (0.6)	6815 (0.4)	0.03
Rural residence	36 804 (17.8)	171 552 (9.1)	0.26
Foreign born	34 349 (16.6)	548 638 (29.2)	0.30
Gravidity, median (IQR)	1.0 (0.0-2.0)	1.0 (0.0-2.0)	0.20
Parity, median (IQR)	1.0 (0.0-1.0)	1.0 (0.0-1.0)	0.01
Within 120 d before index pregnancy			
No. of ADGs <120 d before pregnancy^b^			
0-2	54 111 (26.2)	1 546 227 (82.2)	1.36
3-4	84 722 (41.0)	269 003 (14.3)	0.63
5-6	46 972 (22.7)	56 038 (3.0)	0.62
7-32	20 734 (10.0)	10 304 (0.5)	0.43
Within 365 d before index pregnancy			
Primary care clinician	199 884 (96.8)	1 732 028 (92.1)	0.21
At index birth			
Gestational age, median (IQR)	39.0 (38.0-40.0)	39.0 (38.0-40.0)	0.06
Preterm birth <32 weeks’ gestation	1829 (0.9)	12 450 (0.7)	0.03
Preterm birth <37 weeks’ gestation	15 476 (7.5)	111 059 (5.9)	0.06
Male newborn	105 524 (51.1)	965 970 (51.3)	0.00
Female newborn	101 015 (48.9)	915 602 (48.7)	0.00
Severe neonatal morbidity	13 581 (6.6)	100 264 (5.3)	0.05

Abbreviations: ADG, Aggregated Diagnosis Group; ED, emergency department.

^a^ For a given variable, a standardized difference greater than 0.10 reflects an important difference between groups.

^b^ Using ADGs within the 120 days before the clinical start of the index pregnancy.

### Risk of Infant ED Visit

The rate of infant ED use in the first year of life was higher in those whose mother had used the ED before pregnancy (570 per 1000) than those who had not (388 per 1000), equivalent to an unadjusted RR of 1.39 (95% CI, 1.38-1.40), an adjusted RR of 1.19 (95% CI, 1.18-1.20), and an adjusted ARD of 91.1 per 1000 (95% CI, 88.6-93.6 per 1000) (Table 2). These risk patterns persisted upon stratifying by infant sex, preterm birth less than 37 weeks’ gestation, and the presence of severe neonatal morbidity during the index birth hospitalization (eTable 2 in Supplement 1). The calculated E-value was 1.67 for the aRR, with a lower 95% CI of 1.64.

**Table 2.** Risk of ED Utilization by a Singleton Infant in the First 365 Days After the Birth Discharge Date by Maternal ED Visit Within 90 Days Preceding the Estimated Conception Date

Exposure	No.	No. of infants with an ED visit (rate per 1000)	Relative risk (95% CI)		Risk difference per 1000 (95% CI)	
			Unadjusted	Adjusted^a^	Unadjusted	Adjusted^a^
No maternal prepregnancy ED visit	1 881 572	730 025 (388.0)	1 [Reference]	1 [Reference]	1 [Reference]	1 [Reference]
Maternal prepregnancy ED visit	206 539	117 614 (569.5)	1.39 (1.38-1.40)	1.19 (1.18-1.20)	155.0 (152.7-157.2)	91.1 (88.6-93.6)

Abbreviation: ED, emergency department.

^a^ Adjusted for a mother’s age, area-level income quintile, rural residence, immigrant status, parity—each at the start of the index pregnancy; as well as Johns Hopkins Adjusted Aggregated Diagnosis Groups (0-2, 3-4, 5-6, ≥7) within 120 days before the start of the index pregnancy, and having a primary care clinician within 365 days before the start of the index pregnancy.

In the unadjusted analysis, a dose-response association was seen between the number of maternal prepregnancy ED visits and the risk of any infant ED visit in the first 365 days following the index birth hospitalization discharge (Table 3). There was not a dose-response association observed in the adjusted analysis: compared with mothers without a prepregnancy ED visit, those with 1 visit had an adjusted RR of 1.19 (95% CI, 1.18-1.20), those with 2 visits had an adjusted RR of 1.18 (95% CI 1.17-1.20) and those with 3 or more visits had an adjusted RR of 1.22 (95% CI, 1.20-1.23). A coupling tendency (ie, pairing) was observed between a low-acuity prepregnancy ED visit in the mother and a low acuity visit for her infant in the first year of life (Table 4). For example, relative to mothers without any ED visit before conception, low-acuity maternal ED visits had an adjusted OR of 5.52 (95% CI, 5.16-5.90) for the corresponding infant having a similarly low-acuity ED encounter. This association was numerically much higher than the adjusted OR of 1.43 (95% CI, 1.38-1.49) observed for a concomitantly high-acuity ED visit among a mother before pregnancy, and that of the infant within 1 year after birth (Table 4).

**Table 3.** Risk of Infant ED Utilization in the First 365 Days After Birth Discharge Date by Number of Maternal ED Visits Within 90 Days Preceding the Estimated Conception Date

No. of maternal prepregnancy ED visits	No.	No. of infants with an ED visit (rate per 1000)	Relative risk (95% CI)	
			Unadjusted	Adjusted^a^
No ED visit	1 881 572	730 025 (388.0)	1 [Reference]	1 [Reference]
1	146 350	80 849 (552.4)	1.36 (1.35-1.36)	1.19 (1.18-1.20)
2	40 452	23 781 (587.9)	1.43 (1.42-1.44)	1.18 (1.17-1.20)
3 or more	19 737	12 984 (657.9)	1.57 (1.56-1.59)	1.22 (1.20-1.23)

Abbreviation: ED, emergency department.

^a^ Adjusted for a mother’s age, area-level income quintile, rural residence, immigrant status, parity—each at the start of the index pregnancy; as well as Johns Hopkins Adjusted Aggregated Diagnosis Groups (0-2, 3-4, 5-6, ≥7) within 120 days before the start of the index pregnancy, and having a primary care clinician within 365 days before the start of the index pregnancy.

**Table 4.** Odds of Infant Having a Less or More Urgent ED Visit Within 365 Days After the Birth Discharge Date by Acuity of Maternal ED Visit Within 90 Days Before Conception^a^

Maternal CTAS score at her latest prepregnancy ED visit^b^	No.	CTAS at infant’s first ED visit^b^					
		1-2		3-4		5	
		No. (rate per 1000 livebirths)	aOR (95% CI)^c^	No. (rate per 1000 livebirths)	aOR (95% CI)^c^	No. (rate per 1000 livebirths)	aOR (95% CI)^c^
1-2	20 466	3385 (165.4)	1.43 (1.38-1.49)	7146 (349.2)	1.30 (1.26-1.35)	286 (14.0)	1.32 (1.17-1.49)
3-4	170 762	23 865 (139.8)	1.38 (1.36-1.40)	69 607 (407.6)	1.58 (1.57-1.60)	3444 (20.2)	1.60 (1.53-1.67)
5	14 645	1373 (93.8)	1.13 (1.06-1.20)	6592 (450.1)	1.80 (1.74-1.87)	1356 (92.6)	5.52 (5.16-5.90)

Abbreviations: aOR, adjusted odds ratio; CTAS, Canadian Triage and Acuity System; ED, emergency department.

^a^ No ED visit serves as the referent in these multinomial logistic regression models. If more than 1 ED visit occurred, the mother’s latest visit was used within 90 days before conception, whereas the infant’s first ED visit was used within 1 year after birth discharge.

^b^ A higher CTAS score indicates a less urgent visit (CTAS level 1-2: resuscitation or emergent; CTAS level 3-4: urgent or less urgent; CTAS level 5: nonurgent).

^c^ Adjusted for a mother’s age, area-level income quintile, rural residence, immigrant status, parity—each at the start of the index pregnancy; as well as Johns Hopkins Adjusted Aggregated Diagnosis Groups (0-2, 3-4, 5-6, ≥7) within 120 days before the start of the index pregnancy, and having a primary care clinician within 365 days before the start of the index pregnancy.

The risk of any infant ED encounter was inversely associated with the recorded cumulative amount of time a patient spent in the ED over the 90-day period before pregnancy (Figure). Relative to no maternal ED visit before pregnancy, those with the shortest ED length of stay (decile 1, 1-57 minutes) had the highest adjusted RR of an infant ED encounter (1.27; 95% CI, 1.26-1.28), whereas those in decile 10 (cumulative stay ≥599 minutes) had the lowest adjusted RR of 1.12 (95% CI, 1.10-1.13) (Figure).

**Figure.**
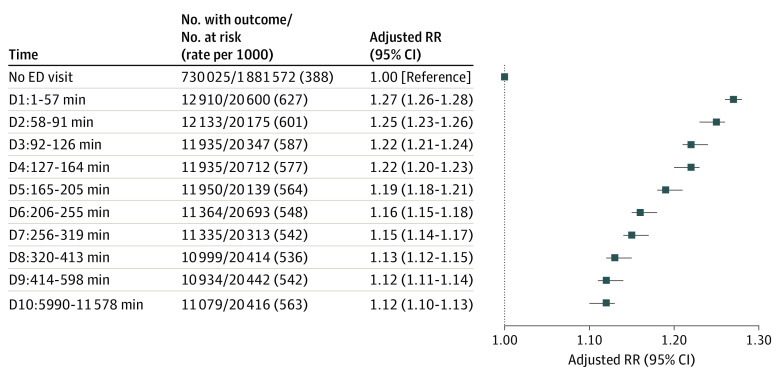
Risk of Emergency Department (ED) Utilization by an Infant in the First 365 Days After Its Birth Hospitalization Discharge Date by Cumulative Number of Minutes of Maternal ED Visits Before Conception Relative risks are adjusted for maternal age, area-level income quintile, rural residence, immigrant status, parity—each at the start of the index pregnancy; as well as Johns Hopkins Adjusted Aggregated Diagnosis Groups (0-2, 3-4, 5-6, ≥7) within 120 days before the start of the index pregnancy, and having a primary care clinician within 365 days before the start of the index pregnancy. D indicates decile.

Relative to no ED visit within 90 days before pregnancy, each of the main diagnostic groups at a patient’s latest prepregnancy ED visit were significantly associated with a higher risk of a subsequent infant ED encounter (eTable 3 in Supplement 1). The highest adjusted RRs were seen for maternal neoplasms (1.34; 95% CI, 1.18-1.51), diseases of the respiratory system (1.32; 95% CI, 1.30-1.33), musculoskeletal system (1.28; 95% CI, 1.26-1.30), and nervous system (1.28; 95% CI, 1.25-1.32).

### Risk of Other Adverse Infant Outcomes

The unadjusted RR risk of infant death within 365 days after the index birth hospitalization discharge date was significantly higher in comparison with maternal prepregnancy ED use (1.45; 95% CI, 1.26-1.65), but not after adjusting for study covariates (1.11; 95% CI, 0.95-1.29) (eTable 4 in Supplement 1). For infant readmission to hospital within 365 days, the corresponding rates were 122 and 90 per 1000 in infants of mothers with vs without prepregnancy ED use (adjusted RR, 1.15; 95% CI, 1.13-1.16) (eTable 4 in Supplement 1). The main diagnosis at an infant’s first ED encounter after birth is listed in eTable 5 in Supplement 1.

## Discussion

About 1 in 10 mothers who gave birth in Ontario had an ED visit within 90 days preceding the start of that index pregnancy, and 4 in 10 infants had an ED visit in their first year after their birth hospital discharge. Prepregnancy maternal ED use was associated with an increased risk of their infant using the ED, especially among mothers with a greater number of prepregnancy ED visits, low-acuity presentations, and a short amount of cumulative time in the ED. No association was seen between preconception maternal ED use and infant mortality after the index birth hospitalization.

### Other Studies

The current study aligns with prior research describing higher in-pregnancy and postpartum ED utilization^12,14,26^ in mothers with comorbidities and other socioeconomic challenges. We previously described some reasons for maternal ED use before pregnancy, which were partly dominated by Main Discharge Diagnosis Groups of “Symptoms, signs, abnormal clinical and laboratory findings,” as well as “Injury, poisonings, and consequences of external causes.“^14^

These findings are also consistent with a select number of considerably smaller studies showing an association between maternal and infant ED use in highly selective populations.^11,15^ For example, among a cohort of 4112 Medicaid-insured mother-infant pairs attending a regional perinatal referral center in Delaware, the number of maternal ED visits was associated with infant ED use 6 months after birth, as were the number of maternal medications, young maternal age, and Black race.^11^ In that study, 11% of infants were rehospitalized, and 41% used the ED within 6 months of birth—rates similar to the current study.

Although the finding that low-acuity maternal ED visits are most correlated with low-acuity infant ED visits may seem counterintuitive, previous studies showed that infants not only have the highest rate of ED use among those under age 18 years, but that those ED visits are dominated by low-acuity issues.^9,10^ Moreover, infants appear twice as likely as other children to have high-frequency, low-resource intensity ED visits, many of which are avoidable.^10^ Given that the mother is often most charged with bringing their child to the ED, a prior tendency to visit the ED for themself could mean the same for their infant. Hence, based on our findings, we now posit that the pattern of low-acuity prepregnancy ED use by a mother reemerges as low-acuity ED access for their infant—a tendency that could be mitigated by new strategies.

### Clinical and Policy Implications

The first year of an infant’s life is a time of higher acute care utilization.^4,7^ There exist a handful of studies focused on reducing frequent ED use for nonurgent pediatric ED visits, with inconclusive findings.^27^ In a randomized clinical trial (RCT) of 1300 families in 2 safety-net health systems in California, provision of an in-person resource navigator significantly decreased the risk of child hospitalization during a 1-year period as compared with the provision of written information.^28^ Even so, ED utilization did not decrease in that study, suggesting that a different strategy might be needed to reduce infant ED use, if effective at all. In an RCT of intensive home visits among 244 families in New Mexico with a first-born child, infants in the treatment group were 33% less likely to visit the ED compared with the families not receiving in-home visits, with no differences in rates of hospitalization or injury.^29^

Given the findings of the current study, future research should determine if, and how, identifying a woman with a recent history of an ED visit for a minor indication may curtail avoidable ED visits for their infant. Future research should evaluate both a woman’s obstetrical and primary care during pregnancy, and that of the newborn, as well as the use of social services after birth, and how they respectively correlate with ED use before and after pregnancy.

### Limitations

This study had limitations. Details were lacking about the pattern of care a mother or infant received within the ED or thereafter, such as the comprehensiveness of maternal prenatal care or infant primary care. Whether a pregnancy was planned was also not known, and midwifery homebirths were not included herein. The travel distance between their home and the nearest ED or location of their primary care provider was not known for a mother or the infant—each being factors that may influence excess ED use.^30^ In addition, while having a primary care clinician within 1 year before conception was considered as a covariate for each mother, the number and nature of obstetrical and primary care visits were not, nor was primary care provision for the newborn.

Pregnancy dating in Ontario is accurately captured for most mothers who give birth.^21^ By setting time zero at 2 weeks before conception, we maximized the likelihood that any prepregnancy ED encounter preceded embryogenesis, and we further adjusted for maternal comorbidities. The fact that this study included all women in Ontario afforded care under a single health care system reduced participant selection bias or information bias. Despite adjusting for certain maternal factors, including antecedent comorbidities, the potential remains for residual confounding between prepregnancy ED use and risk of infant ED encounters. For example, although we accounted for area-level income status, rurality and immigrant status, ethnicity was not known. Even so, the calculated E-value of 1.67, and its lower 95% CI of 1.64, suggest that a relatively high degree of unmeasured confounding would need to be present to fully explain away the observed association between maternal ED use before pregnancy and infant ED use in the first year of life, if assumed to be causal in nature.

To assess ED use in infancy, this study design required that a newborn survived to hospital discharge after their birth. For this reason, infant mortality was assessed from the initial discharge date from hospital up to 365 days thereafter, potentially leading to lower-than-expected infant mortality rates. Even so, the current study did assess both subsequent ED use and infant rehospitalization.

## Conclusions

In this cohort study of singleton livebirths, prepregnancy ED use was common among mothers, as was ED use in infancy. Prepregnancy ED use was associated with a higher rate of ED use in an infant’s first year, which was largely not explained by maternal or infant illness. This paired pattern may potentially offer a useful trigger for health system interventions designed to decrease acute care utilization for low-acuity indications in infancy.

## Notes

Supplement 1. **eFigure.** Flow Diagram of Cohort Creation **eTable 1.** Variables Used to Define Cohort Entry and Exclusion Criteria, Outcomes, and Adjustment Variables **eTable 2.** Stratification of Analysis 1 by the Infant’s Sex, Timing of Birth, and the Presence of Severe Neonatal Morbidity During Its Index Birth Hospitalization **eTable 3.** Risk of the Primary Study Outcome of Emergency Department (ED) Utilization by a Singleton Infant in the First 365 Days After Birth and Survival to Hospital Discharge, in Relation to the Main Discharge Diagnosis Group at the Latest (Most Recent) Preconception Emergency Department (ED) Visit Within 90 Days Before Conception **eTable 4.** Risk of the Secondary Study Outcomes of (I) Infant Death Within 365 Days After the Index Birth Hospital Discharge; (II) Re-admission to Hospital Within 365 Days After the Index Birth Hospital Discharge – Each in Relation to a Woman Having an Emergency Department (ED) Visit(s) Within 90 Days Preceding the Estimated Conception Date **eTable 5.** Main Discharge Diagnosis at the Infant’s First ED Encounter. All Data Are Presented as a Number (%), Where the Denominator is Among All Infants Who Had a First ED Encounter

Supplement 2.
Data Sharing Statement
