# Supplementary material for: Maternal Emergency Department Use Before Pregnancy and Infant Emergency Department Use After Birth
Source: JAMA Netw Open. 2023 Mar 13;6(3):e232931. doi: 10.1001/jamanetworkopen.2023.2931 (PMC10011931; doi:10.1001/jamanetworkopen.2023.2931)
Supplement: Supplement 1. — eFigure. Flow Diagram of Cohort Creation eTable 1. Variables Used to Define Cohort Entry and Exclusion Criteria, Outcomes, and Adjustment Variables eTable 2. Stratification of Analysis 1 by the Infant’s Sex, Timing of Birth, and the Presence of Severe Neonatal Morbidity During Its Index Birth Hospitalization eTable 3. Risk of the Primary Study Outcome of Emergency Department (ED) Utilization by a Singleton Infant in the First 365 Days After Birth and Survival to Hospital Discharge, in Relation to the Main Discharge Diagnosis Group at the Latest (Most Recent) Preconception Emergency Department (ED) Visit Within 90 Days Before Conception eTable 4. Risk of the Secondary Study Outcomes of (I) Infant Death Within 365 Days After the Index Birth Hospital Discharge; (II) Re-admission to Hospital Within 365 Days After the Index Birth Hospital Discharge – Each in Relation to a Woman Having an Emergency Department (ED) Visit(s) Within 90 Days Preceding the Estimated Conception Date eTable 5. Main Discharge Diagnosis at the Infant’s First ED Encounter. All Data Are Presented as a Number (%), Where the Denominator is Among All Infants Who Had a First ED Encounter [file jamanetwopen-e232931-s001.pdf]

Supplemental Online Content

Varner CE, Park AL, Ray JG. Maternal emergency department use before pregnancy and infant emergency department use after birth. *JAMA Netw Open.* 2023;6(3):e232931. doi:10.1001/jamanetworkopen.2023.2931

eFigure. Flow Diagram of Cohort Creation

eTable 1. Variables Used to Define Cohort Entry and Exclusion Criteria, Outcomes, and Adjustment Variables

eTable 2. Stratification of Analysis 1 by the Infant's Sex, Timing of Birth, and the Presence of Severe Neonatal Morbidity During Its Index Birth Hospitalization

eTable 3. Risk of the Primary Study Outcome of Emergency Department (ED) Utilization by a Singleton Infant in the First 365 Days After Birth and Survival to Hospital Discharge, in Relation to the Main Discharge Diagnosis Group at the Latest (Most Recent) Preconception Emergency Department (ED) Visit Within 90 Days Before Conception

eTable 4. Risk of the Secondary Study Outcomes of (I) Infant Death Within 365 Days After the Index Birth Hospital Discharge; (II) Re-admission to Hospital Within 365 Days After the Index Birth Hospital Discharge – Each in Relation to a Woman Having an Emergency Department (ED) Visit(s) Within 90 Days Preceding the Estimated Conception Date

eTable 5. Main Discharge Diagnosis at the Infant's First ED Encounter. All Data Are Presented as a Number (%), Where the Denominator is Among All Infants Who Had a First ED Encounter

This supplemental material has been provided by the authors to give readers additional information about their work.

© 2023 Varner CE et al. *JAMA Network Open.*

eFigure. Flow diagram of cohort creation

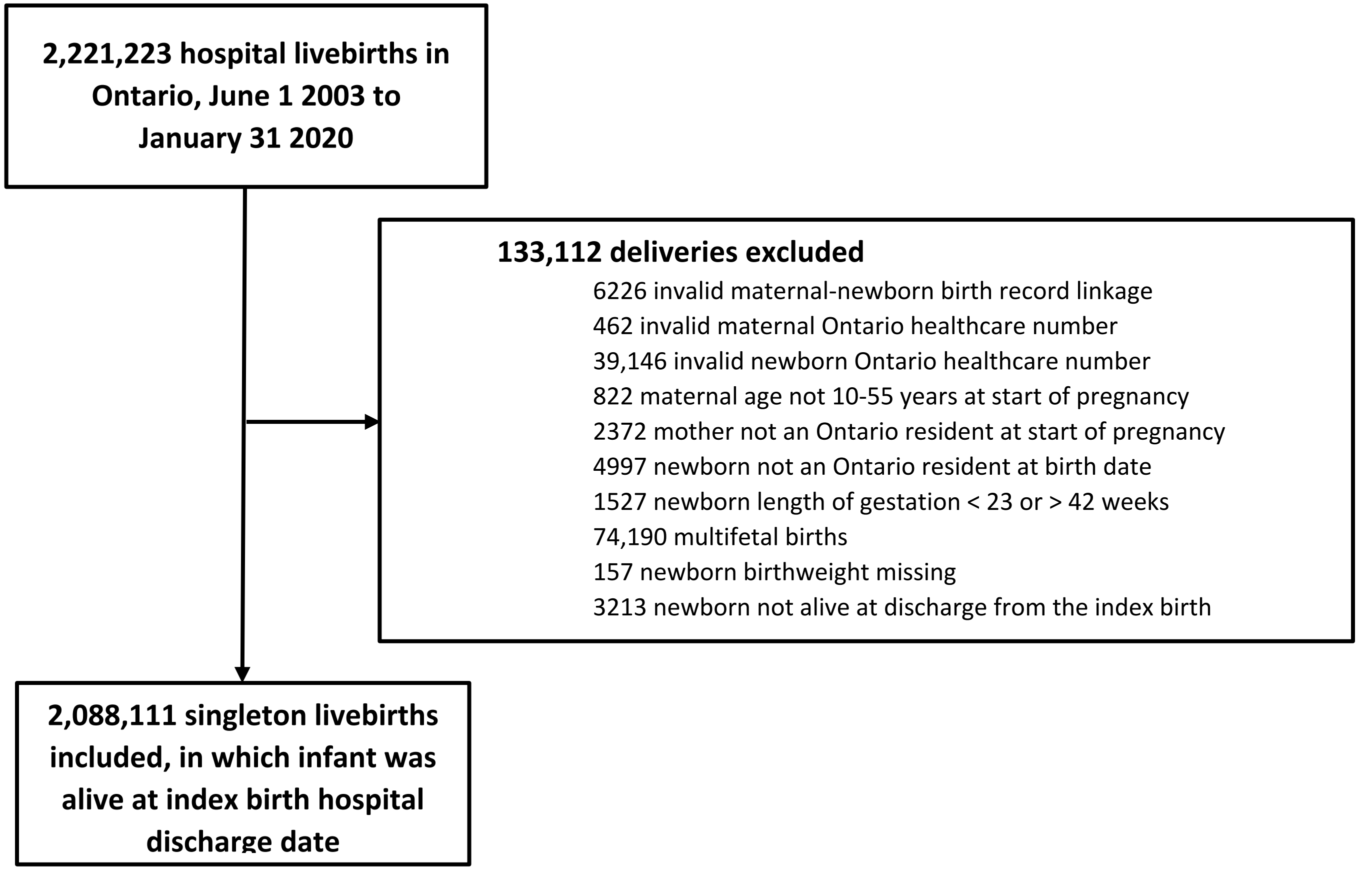

© 2023 Varner CE et al. *JAMA Network Open.*

eTable 1. Variables used to define cohort entry and exclusion criteria, outcomes and adjustment variables

Assessment	Timing	Disease, procedure or measure	ICD-10-CA diagnostic codes and CCI procedure codes [bolded], in DAD, SDS and NACRS	ICD-9 diagnostic codes in OHIP {or other sources}
Cohort entry criteria	At the index delivery date (where the estimated clinical start of pregnancy [i.e. 0 weeks' gestation] is June 1, 2003 to January 31, 2020)	Women in Ontario with a singleton livebirth, in which the liveborn child survived to hospital discharge, from June 1, 2003 to January 31, 2020.	Main patient service code indicating "obstetrical delivery" (the MOMBABY dataset includes linked DAD inpatient admission records of delivering mothers and their newborns; https://datadictionary.ices.on.ca/Applications/DataDictionary/Library.aspx?Library=MOMBABY)	--
Exclusion criteria	From 0 weeks' gestation up to and including 42 days' postpartum	Woman had an invalid healthcare number or hospital number	Invalid healthcare number on the DAD delivery record, or invalid maternal-newborn linkage in MOMBABY	Sex not 'Female', birth date missing, or unregistered healthcare number {RPDB}
	Same as above	Woman was a non-Ontario resident at any time during the perinatal period	--	Postal code {RPDB}
	Same as above	Woman was not OHIP eligible during the entire perinatal period	--	Eligibility start and end dates {RPDB}
	Estimated clinical start of pregnancy	Woman's recorded death date preceded pregnancy	--	Death date {RPDB}

© 2023 Varner CE et al. *JAMA Network Open.*

Assessment	Timing	Disease, procedure or measure	ICD-10-CA diagnostic codes and CCI procedure codes [bolded], in DAD, SDS and NACRS	ICD-9 diagnostic codes in OHIP {or other sources}
	Same as above	Woman's age was missing or < 10 or > 55 years	--	Age {RPDB}
	At the index birth	Length of gestation missing, < 20 weeks or > 42 weeks	Clinical gestation weeks at delivery (from the DAD newborn record, if present, otherwise from the DAD delivery record)	--
	Same as above	Multifetal delivery	Z37.2-Z37.7, Z37.90, O30, O31 (DAD delivery record) or Z38.3-Z38.6, Q89.4 (DAD newborn record)	--
	Same as above	Liveborn infant with unknown birthweight	Newborn weight (DAD newborn record)	--
	Same as above	Liveborn infant discharged alive and had an invalid healthcare number	Discharged alive and invalid healthcare number (DAD newborn record)	Sex missing, birth date missing, or unregistered healthcare number {RPDB}
Main Exposure	Within 90 days before the estimated clinical start of pregnancy	ED visit during the pre-pregnancy period	Any ICD-10-CA code in NACRS	--
Secondary Exposures	Within 90 days before the estimated clinical start of pregnancy	Cumulative time spent in the ED (ED length of stay)	NACRS	--
	Same as above	ED triage acuity as measured by the CTAS	NACRS	--

© 2023 Varner CE et al. *JAMA Network Open.*

Assessment	Timing	Disease, procedure or measure	ICD-10-CA diagnostic codes and CCI procedure codes [bolded], in DAD, SDS and NACRS	ICD-9 diagnostic codes in OHIP {or other sources}
	Same as above	Main diagnostic code for the ED visit	Any ICD-10-CA code in NACRS	--
Stratification Variables	At the index birth	Preterm birth < 32 weeks' gestation	Pre-term deliveries (DAD delivery record)	--
	Same as above	Preterm birth < 37 weeks' gestation	Pre-term deliveries (DAD delivery record)	--
	Same as above	Infant sex	--	Sex {RPDB}

© 2023 Varner CE et al. *JAMA Network Open.*

	Arising < 28 days after a livebirth	Severe neonatal morbidity (SNM), using the severe neonatal adverse outcomes indicator (NAOI)	**All indicators are identified from the DAD newborn record:**<br><br>Gestational age at birth < 32 weeks<br><br>Birthweight < 1500 grams<br><br>Respiratory distress syndrome: P22.0<br><br>Seizures: P90, R56<br><br>Intraventricular haemorrhage (grades 3 and 4): P52.2<br><br>Cerebral infarction: I63<br><br>Periventricular leukomalacia: P91.2<br><br>Birth Trauma (intracranial hemorrhage paralysis due to brachial plexus injury, skull or long bone fracture): P10.0-3, P13.0, P13.2-3, P14.0-1<br><br>Hypoxic ischemic encephalopathy: P91.5, P91.8, P91.6<br><br>Necrotising enterocolitis: P77<br><br>Sepsis/septicaemia (streptococcus staphylococcus, E.coli, unspecified gram negative): P36.0-8, B95.1, B96.2<br><br>Pneumonia: P23, J12-18<br><br>Other respiratory (primary atelectasis, respiratory failure): P28.0, P28.5<br><br>Chronic respiratory disease originating in the perinatal period: P27<br><br>Bacterial meningitis: G00-03, G05<br><br>Resuscitation: 1.GZ.30.CJ, 1.GZ.30.CJ-NB, 1.GZ.30.JH, 1.HZ.30.JN, 1.HZ.30.JY	Hypothermia (therapeutic) induction G210

© 2023 Varner CE et al. *JAMA Network Open.*

			Ventilatory support (mechanical ventilation and/or CPAP): 1.GZ.31.CA-EP, 1.GZ.31.CA-ND, 1.GZ.31.CA-PK, 1.GZ.31.CB-ND, 1.GZ.31.CR-ND, 1.GZ.31.GP-ND, 1.GZ.31.JA-GX, 1.GZ.31.JA-MD, 1.GZ.31.JA-NC, 1.GZ.31.JA-PK<br><br>Central venous or arterial catheter: 1.KV.53.HA-CH, 1.KV.53.HA-FT, 1.KV.53.LA-FT, 2.IM.28.GP, 2.LZ.28.GQ-PL, 2.LZ.28.GR-PL, 2.LZ.28.JA-PL, 1.KX.53.HA-CH, 1.KX.53.HA-FT, 1.KX.53.LA-FT, 2.LZ.28.GQ-PL, 2.LZ.28.GR-PL<br><br>Pneumothorax requiring intercostal catheter: P25.1, or 1.GV.52.DA, 1.GV.52.DA-TS, 1.GV.52.HA, 1.GV.52.HA-HE, 1.GV.52.HA-TK, 1.GV.52.LA, 1.GV.52.LA-TS, 1.GV.52.LA-XX-E, 1.GV.54.JA-TS, 1.GV.55.JA-TS<br><br>Any intravenous fluids: 1.LZ.35.CA-E6, 1.LZ.35.HA-C1, 1.LZ.35.HA-C5, 1.LZ.35.HA-C6, 1.LZ.35.HA-C7, 1.LZ.35.HA-E6, 1.LZ.35.HA-T7, 1.LZ.35.HA-T9, 1.LZ.35.HA-Z9, 1.LZ.35.HH-C1, 1.LZ.35.HH-C5, 1.LZ.35.HH-C6, 1.LZ.35.HH-C7, 1.LZ.35.HH-E0, 1.LZ.35.HH-E6, 1.LZ.35.HH-T7, 1.LZ.35.HH-T9, 1.LZ.35.HH-Z9, 1.LZ.35.HR-C5, 1.LZ.35.HR-C6, 1.LZ.35.HR-C7, 1.LZ.35.HR-T9, 1.LZ.35.HR-Z9<br><br>Any body cavity surgical procedure: 1.AA.52, 1.AA.87, 1.AC.87, 1.AE.87, 1.AF.87, 1.AG.87, 1.AJ.87, 1.AK.87, 1.AN.52, 1.AN.59, 1.AN.87, 1.AP.59, 1.AP.72, 1.AP.87, 1.AW.59, 1.AW.72, 1.AW.87, 1.AX.87, 1.BA.72, 1.BA.80, 1.BA.87, 1.BB.72, 1.BB.80, 1.BB.87, 1.BD.72, 1.BD.80, 1.BD.87, 1.BF.80, 1.BG.72, 1.BG.80, 1.BG.87, 1.BK.59, 1.BM.72, 1.BM.80, 1.BM.87, 1.BN.72, 1.BN.80, 1.BN.87, 1.BP.72, 1.BP.80, 1.BP.87, 1.BQ.72, 1.BQ.80, 1.BQ.87, 1.BS.72, 1.BS.80, 1.BS.87, 1.BT.72, 1.BT.80, 1.BT.87, 1.GA.87, 1.GA.89, 1.GB.87, 1.GB.89, 1.GD.89, 1.GE.80, 1.GE.87, 1.GE.89, 1.GE.91, 1.GH.84, 1.GJ.86, 1.GJ.87, 1.GK.87, 1.GK.89, 1.GM.80, 1.GM.86, 1.GM.87, 1.GN.92, 1.GR.87, 1.GR.89, 1.GR.91, 1.GT.78, 1.GT.87, 1.GT.89, 1.GT.91, 1.GV.87, 1.GV.89, 1.GW.87, 1.GX.80, 1.GX.86, 1.GX.87, 1.GY.70, 1.GY.72, 1.GY.86, 1.HJ.76, 1.HJ.82, 1.HN.87, 1.HP.76, 1.HP.78, 1.HP.80, 1.HP.82, 1.HP.83, 1.HP.87, 1.HR.80, 1.HR.84, 1.HR.87, 1.HS.80 (excl. 1.HS.80.G), 1.HS.90, 1.HT.80 (excl. 1.HT.80.G), 1.HT.89, 1.HT.90, 1.HU.80 (excl. 1.HU.80.G), 1.HU.90, 1.HV.80 (excl. 1.HV.80.G), 1.HV.90, 1.HW.78, 1.HW.79, 1.HX.80, 1.HX.87, 1.HX.80, 1.HZ.87, 1.IA.76, 1.IA.80, 1.IA.87, 1.IB.76, 1.IB.79, 1.IB.80, 1.IB.82, 1.IB.87, 1.IC.76, 1.IC.80, 1.IC.82, 1.IC.87, 1.ID.76, 1.ID.80, 1.ID.82, 1.ID.86, 1.ID.87, 1.IF.83, 1.IJ.76, 1.IJ.80, 1.IM.76, 1.IM.80, 1.IM.82, 1.IM.83, 1.IM.87, 1.IN.83, 1.IN.84, 1.IN.87, 1.JE.57 (excl. 1.JE.57.G), 1.JE.76,	

© 2023 Varner CE et al. *JAMA Network Open.*

Assessment	Timing	Disease, procedure or measure	ICD-10-CA diagnostic codes and CCI procedure codes [bolded], in DAD, SDS and NACRS	ICD-9 diagnostic codes in OHIP {or other sources}
			1.JE.80, 1.JE.87, 1.JJ.76, 1.JJ.80, 1.JK.76, 1.JK.80, 1.JK.87, 1.JW.51 (excl. 1.JW.51.G), 1.JW.57, 1.JW.76, 1.LA.84, 1.LC.84, 1.LD.84, 1.NA.72, 1.NA.74, 1.NA.76, 1.NA.77, 1.NA.80, 1.NA.84, 1.NA.86, 1.NA.87, 1.NA.88, 1.NA.89, 1.NA.90, 1.NA.91, 1.NA.92, 1.NE.80, 1.NF.76, 1.NF.78, 1.NF.80, 1.NF.82, 1.NF.84, 1.NF.86, 1.NF.87 (excl. 1.NF.87.B), 1.NF.89, 1.NF.90, 1.NF.91, 1.NF.92, 1.NK.76, 1.NK.77, 1.NK.80, 1.NK.82, 1.NK.84, 1.NK.87 (excl. 1.NK.87.B), 1.NM.74, 1.NM.76, 1.NM.77, 1.NM.80, 1.NM.82, 1.NM.87 (excl. 1.NM.87.B), 1.NM.89, 1.NM.91, 1.NP.72, 1.NP.73, 1.NP.86, 1.NQ.74 (excl. 1.NQ.74.B), 1.NQ.80, 1.NQ.84, 1.NQ.86, 1.NQ.87 (excl. 1.NQ.87.B), 1.NQ.89, 1.NQ.90, 1.NT.80, 1.NT.84, 1.NT.86, 1.NT.87, 1.NV.89, 1.OA.87, 1.OB.87, 1.OB.89, 1.OD.76, 1.OD.89, 1.OE.76, 1.OE.80, 1.OE.89, 1.OJ.76 (excl. 1.OJ.76.B), 1.OJ.87, 1.OJ.89, 1.OK.87, 1.OK.89, 1.OK.91, 1.OT.72, 1.OT.87, 1.OT.91, 1.PB.87, 1.PB.89, 1.PC.80, 1.PC.87 (excl. 1.PC.87.D), 1.PC.89, 1.PC.91, 1.PE.57 (excl. 1.PE.57.BD), 1.PE.80 (excl. 1.PE.80.D), 1.PE.82, 1.PE.87 (excl. 1.PE.87.D), 1.PE.89 (excl. 1.PE.89.D), 1.PG.76, 1.PG.77, 1.PG.80 (excl. 1.PG.80.D), 1.PG.86, 1.PG.89, 1.PL.74 (excl. 1.PL.74.CD), 1.PL.80, 1.PM.79, 1.PM.86, 1.PM.87 (excl. 1.PM.87.B), 1.PM.89, 1.PM.90, 1.PM.91, 1.PM.92, 1.QE.53, 1.QE.80, 1.QE.82, 1.QE.84, 1.QE.87, 1.QE.89, 1.QG.89, 1.QM.74, 1.QM.80, 1.QM.87, 1.QM.89, 1.QM.91, 1.QN.82, 1.QT.87, 1.QT.91, 1.RB.74, 1.RB.80, 1.RB.83, 1.RB.87, 1.RB.89, 1.RD.89, 1.RF.51, 1.RF.72, 1.RF.74, 1.RF.80, 1.RF.87, 1.RF.89, 1.RM.87 (excl. 1.RM.87.B), 1.RM.89, 1.RM.91, 1.RN.87, 1.RN.89, 1.RS.74, 1.RS.80, 1.RS.86, 1.RS.87, 1.RS.89, 1.RW.87, 1.RW.88, 1.RW.91, 1.RW.92, 1.SA.74, 1.SA.75, 1.SA.80, 1.SA.89, 1.SC.74, 1.SC.75, 1.SC.80, 1.SC.87, 1.SC.89, 1.SE.53, 1.SE.89 (excl. 1.SE.89.D), 1.SF.80, 1.SF.87, 1.SF.89, 1.SG.80, 1.SG.87, 1.SH.87, 1.SM.74, 1.SM.80, 1.SM.87, 1.SN.87, 1.SN.93, 1.SQ.53, 1.SQ.74, 1.SQ.80, 1.SQ.87, 1.SQ.91, 1.SQ.93, 1.SW.74, 1.SY.80, 1.SY.84, 1.SY.87, 1.SZ.87, 1.VA.53, 1.VA.74, 1.VA.75, 1.VA.80, 1.VA.87, 1.VA.93, 1.VC.74, 1.VC.80, 1.VC.87, 1.VC.91, 1.VC.93, 1.VE.80, 1.VG.53, 1.VG.55, 1.VG.72, 1.VG.73, 1.VG.74, 1.VG.75, 1.VG.80, 1.VG.87, 1.VG.93, 1.VK.80, 1.VK.87, 1.VK.89, 1.VL.80, 1.VL.87, 1.VM.80, 1.VM.87, 1.VN.80, 1.VN.87, 1.VP.74, 1.VP.80, 1.VP.87, 1.VP.89, 1.VQ.74, 1.VQ.79, 1.VQ.80, 1.VQ.82, 1.VQ.87, 1.VQ.91, 1.VQ.93, 1.VS.72, 1.VS.80, 1.VX.87	

© 2023 Varner CE et al. *JAMA Network Open.*

Assessment	Timing	Disease, procedure or measure	ICD-10-CA diagnostic codes and CCI procedure codes [bolded], in DAD, SDS and NACRS	ICD-9 diagnostic codes in OHIP {or other sources}
Primary outcome	Within 365 days after the index birth hospitalization discharge date	Any infant ED visit	Any ICD-10-CA code in NACRS	--
Secondary outcomes	Within 365 days after the index birth hospitalization discharge date	Infant death after index birth hospital discharge	--	Death date {RPDB}
	Same as above	Infant re-admission to hospital	CIHI-DAD	--
	Same as above	Infant ED triage acuity measured by the CTAS score, confined to their first ED visit	NACRS	--
	Same as above	Main diagnostic code for the infant ED visit, confined to their first ED visit	NACRS	--
Covariates	At the estimated clinical start of the pregnancy	Woman's age	--	Age {RPDB}
	Same as above	Woman's area-level income quintile	--	{Statistics Canada census}
	Same as above	Woman's rural residence	--	{Statistics Canada census}

© 2023 Varner CE et al. *JAMA Network Open.*

Assessment	Timing	Disease, procedure or measure	ICD-10-CA diagnostic codes and CCI procedure codes [bolded], in DAD, SDS and NACRS	ICD-9 diagnostic codes in OHIP {or other sources}
	Same as above	Woman's immigrant status	--	{IRCC Permanent Resident Database}
	At the index delivery	Woman's number of previous deliveries	Previous term deliveries + Previous pre-term deliveries (DAD delivery record)	--
	Same as above	Woman's number of previous pregnancies	Previous term deliveries + Previous pre-term deliveries + Previous spontaneous abortions + Previous therapeutic abortions (DAD delivery record)	--
	Within 120 days before the estimated clinical start of pregnancy	Total number of Aggregated Diagnosis Groups (ADGs; 0-2, 3-4, 5-6, 7-32)	ADGs were obtained from diagnosis codes in DAD, SDS and NACRS using The Johns Hopkins ACG® System Version 10	--
	Within 365 days before pregnancy	Primary care provider	--	ICES "Primary Care Provider Assignment" algorithm {CAPE, OHIP, ESTSOB}

ACG Adjusted Clinical Group; ADG Aggregated Diagnosis Group; CAPE Client Agency Program Enrolment; CCI Canadian Classification of Interventions; CCP Canadian Classification of Diagnoses and Procedures; ESTSOB Estimated Schedule of Benefits; ICD-9 International Classification of Diseases, 9th Revision; ICD-10-CA International Classification of Diseases, 10th Revision, Canada; NACRS National Ambulatory Care Reporting System; OHIP Ontario Health Insurance Plan; RPDB Registered Persons Database; SDS Same Day Surgery Database

© 2023 Varner CE et al. *JAMA Network Open.*

eTable 2. Stratification of the risk of infant ED use in the first year of life in relation to a woman having any vs. no ED visit within 90 days before pregnancy by the infant's sex, timing of birth, and the presence of severe neonatal morbidity during its index birth hospitalization.

			Relative Risk (95% Confidence Interval)	
**Stratification by infant's characteristic at its birth**	***Exposure:*** **Maternal ED visit(s) within 90 d before conception[a]**	***Outcome:*** **Number of infants with an ED visit (rate per 1000)**	**Unadjusted**	**Adjusted[b]**
Female (N = 1,016,617)	No ED Visit (N = 915,602)	334,486 (365.3)	1.00 (ref.)	1.00 (ref.)
	ED Visit (N = 101,015)	55,351 (547.9)	1.46 (1.45-1.47)	1.22 (1.21-1.23)
Male (N = 1,071,494)	No ED Visit (N = 965,970)	395,539 (409.5)	1.00 (ref.)	1.00 (ref.)
	ED Visit (N = 105,524)	62,263 (590.0)	1.40 (1.39-1.41)	1.19 (1.18-1.20)
Term birth at 37+ weeks' gestation (N = 1,961,576)	No ED Visit (N = 1,770,513)	678,333 (383.1)	1.00 (ref.)	1.00 (ref.)
	ED Visit (N = 191,063)	107,564 (563.0)	1.40 (1.39-1.40)	1.19 (1.19-1.20)
Preterm birth at < 37 weeks' gestation (N = 126,535)	No ED Visit (N = 111,059)	51,692 (465.4)	1.00 (ref.)	1.00 (ref.)
	ED Visit (N = 15,476)	10,050 (649.4)	1.38 (1.36-1.40)	1.17 (1.15-1.18)
Severe neonatal morbidity absent (N = 1,974,266)	No ED Visit (N = 1,781,308)	680,685 (382.1)	1.00 (ref.)	1.00 (ref.)
	ED Visit (N = 192,958)	108,697 (563.3)	1.40 (1.39-1.40)	1.19 (1.19-1.20)
Severe neonatal morbidity present (N = 113,845)	No ED Visit (N = 100,264)	49,340 (492.1)	1.00 (ref.)	1.00 (ref.)
	ED Visit (N = 13,581)	8917 (656.6)	1.33 (1.31-1.35)	1.15 (1.13-1.17)

[a] In these analyses, a woman may have had more than one ED visit before the index pregnancy.

[b]Adjusted for a woman's age, area-level income quintile, rural residence, immigrant status, parity -- each at the start of the index pregnancy; as well as Johns Hopkins Adjusted Aggregated Diagnosis Groups (0-2, 3-4, 5-6, 7+) within 120 d before the start of the index pregnancy, and having a primary care provider within 365 d before the start of the index pregnancy.

© 2023 Varner CE et al. *JAMA Network Open.*

eTable 3. Risk of the primary study outcome of emergency department (ED) utilization by a singleton infant in the first 365 days after birth and survival to hospital discharge, in relation to the main discharge diagnosis group at the latest (most recent) preconception emergency department (ED) visit within 90 days before conception. This analysis compares 206,539 women who had a preconception ED visit with each given discharge diagnosis to 1,881,572 women who did not have any preconception ED visit (referent).

	Relative Risk (95% CI)	
**Main discharge diagnosis group at the mother's most recent ED visit preceding conception**	**Unadjusted**	**Adjusted[a]**
No ED visit within 90 days before pregnancy	1,881,572	1.00 (ref.)
Infections and parasitic diseases (A00-B99)	6439	1.49 (1.46-1.52)
Neoplasms (C00-D48)	189	1.46 (1.29-1.66)
Diseases of the blood and blood-forming organs (D50-D89)	231	1.37 (1.22-1.55)
Endocrine, nutritional, and metabolic diseases (E00-E90)	539	1.55 (1.45-1.65)
Mental, behavioral and neurodevelopmental disorders (F00-F99)	5696	1.50 (1.47-1.53)
Diseases of the nervous system (G00-G99)	3236	1.52 (1.48-1.56)
Diseases of the circulatory system (I00-I99)	1031	1.31 (1.24-1.39)
Diseases of the respiratory system (J00-J99)	18,641	1.56 (1.54-1.58)
Diseases of the digestive system (K00-K93)	10,014	1.44 (1.42-1.46)
Diseases of the skin and subcutaneous tissue (L00-L99)	4703	1.48 (1.45-1.52)
Diseases of the musculoskeletal system (M00-M99)	8707	1.49 (1.46-1.52)
Diseases of the genitourinary system (N00-N99)	22,934	1.45 (1.44-1.47)
Pregnancy (O00-O99)	40,157	1.14 (1.13-1.15)
Symptoms, signs, abnormal clinical and laboratory findings, NEC (R00-R99)	34,062	1.46 (1.44-1.47)
Injury, poisonings, and consequences of external causes (S00-T98)	32,232	1.38 (1.36-1.39)
Other or unknown diagnosis	17,728	1.41 (1.40-1.43)

[a]Adjusted for a woman's age, area-level income quintile, rural residence, immigrant status, parity -- each at the start of the index pregnancy; as well as Johns Hopkins Adjusted Aggregated Diagnosis Groups (0-2, 3-4, 5-6, 7+) within 120 days before the start of the index pregnancy, and having a primary care provider within 365 days before the start of the index pregnancy.

© 2023 Varner CE et al. *JAMA Network Open.*

eTable 4. Risk of the secondary study outcomes of (i) Infant death within 365 days after the index birth hospital discharge; (ii) Re-admission to hospital within 365 days after the index birth hospital discharge – each in relation to a woman having an emergency department (ED) visit(s) within 90 days preceding the estimated conception date.

			Relative Risk (95% Confidence Interval)	
**Maternal ED visit within 90 d before conception**	**Outcome**	**Number of outcome events (rate per 1000)**	**Unadjusted**	**Adjusted[b]**
No ED Visit (N = 1,881,572)	Infant death after index birth hospital discharge	1638 (0.9)	1.00 (ref.)	1.00 (ref.)
ED Visit (N = 206,539)		263 (1.3)	1.45 (1.26-1.65)	1.11 (0.95-1.29)
No ED Visit (N = 1,881,572)	Infant hospital readmission after birth hospital discharge	168,948 (89.8)	1.00 (ref.)	1.00 (ref.)
ED Visit (N = 206,539)		25,229 (122.2)	1.34 (1.32-1.36)	1.15 (1.13-1.16)

[a]In these analyses, a woman may have had more than one ED visit before the index pregnancy.

[b]Adjusted for a woman's age, area-level income quintile, rural residence, immigrant status, parity -- each at the start of the index pregnancy; as well as Johns Hopkins Adjusted Aggregated Diagnosis Groups (0-2, 3-4, 5-6, 7+) within 120 days before the start of the index pregnancy, and having a primary care provider within 365 days before the start of the index pregnancy.

© 2023 Varner CE et al. *JAMA Network Open.*

eTable 5. Main discharge diagnosis at the infant's first ED encounter. All data are presented as a number (%), where the denominator is among all infants who had a first ED encounter.

Main ED discharge diagnosis at infant's first ED encounter after birth (ICD-10-CA codes)	Number (%)
Infections and parasitic diseases (A00-B99)	88,108 (10.4)
Neoplasms (C00-D48)	873 (0.1)
Diseases of the blood and blood-forming organs (D50-D89)	773 (0.3)
Endocrine, nutritional, and metabolic diseases (E00-E90)	2645 (0.1)
Mental, behavioral and neurodevelopmental disorders (F00-F99)	504 (0.1)
Diseases of the nervous system (G00-G99)	1612 (0.2)
Diseases of the eye and adnexa (H00-H59)	17,455 (2.1)
Diseases of the ear and mastoid process (H60-H95)	31,005 (3.7)
Diseases of the circulatory system (I00-I99)	1209 (0.1)
Diseases of the respiratory system (J00-J99)	215,981 (25.5)
Diseases of the digestive system (K00-K93)	50,575 (6.0)
Diseases of the skin and subcutaneous tissue (L00-L99)	27,385 (3.2)
Diseases of the musculoskeletal system (M00-M99)	1617 (0.2)
Diseases of the genitourinary system (N00-N99)	14,908 (1.8)
Certain conditions originating in the perinatal period (P00-P96)	84,257 (9.9)
Congenital malformations, deformations, chromosomal abnormalities (Q00-Q99)	5421 (0.6)
Symptoms, signs, abnormal clinical and laboratory findings, NEC (R00-R99)	165,764 (19.6)
Injury, poisonings, and consequences of external causes (S00-T98)	83,845 (9.9)
Provisional codes (U00-U99)	60 (0.0)
Factors influencing health status and contact with health services (Z00-Z99)	53,598 (6.3)
Diagnosis not recorded	44 (0.0)

ICD-10-CA International Statistical Classification of Diseases and Related Health Problems, 10th Revision, enhanced for Canada

© 2023 Varner CE et al. *JAMA Network Open.*
