# Supplementary material for: Maternal Emergency Department Use Before Pregnancy and Infant Emergency Department Use After Birth
Source: JAMA Netw Open. 2023 Mar 13;6(3):e232931. doi: 10.1001/jamanetworkopen.2023.2931 (PMC10011931; doi:10.1001/jamanetworkopen.2023.2931)
Supplement: Supplement 2. — Data Sharing Statement [file jamanetwopen-e232931-s002.pdf]

Data Sharing Statement

Varner. Maternal Emergency Department Use Before Pregnancy and Infant Emergency Department Use After Birth. *JAMA Netw Open*. Published March 13, 2023.
doi:10.1001/jamanetworkopen.2023.2931

Data
Data available: Yes
Data types: Other (please specify)
Additional Information: The dataset from this study is held securely in coded form at ICES. While data sharing agreements prohibit ICES from making the dataset publicly available, access may be granted to those who meet pre-specified criteria for confidential access, available at www.ices.on.ca/DAS. The full dataset creation plan and underlying analytic code are available from the authors upon request, understanding that the computer programs may rely upon coding templates or macros that are unique to ICES and are therefore either inaccessible or may require modification.
How to access data: www.ices.on.ca/DAS
When available: With publication

Supporting Documents
Document types: None

Additional Information
Who can access the data: The dataset from this study is held securely in coded form at ICES. While data sharing agreements prohibit ICES from making the dataset publicly available, access may be granted to those who meet pre-specified criteria for confidential access, available at www.ices.on.ca/DAS. The full dataset creation plan and underlying analytic code are available from the authors upon request, understanding that the computer programs may rely upon coding templates or macros that are unique to ICES and are therefore either inaccessible or may require modification.
Types of analyses: The dataset from this study is held securely in coded form at ICES. While data sharing agreements prohibit ICES from making the dataset publicly available, access may be granted to those who meet pre-specified criteria for confidential access, available at www.ices.on.ca/DAS. The full dataset creation plan and underlying analytic code are available from the authors upon request, understanding that the computer programs may rely upon coding templates or macros that are unique to ICES and are therefore either inaccessible or may require modification.
Mechanisms of data availability: While data sharing agreements prohibit ICES from making the dataset publicly available, access may be granted to those who meet pre-specified criteria for confidential access, available at www.ices.on.ca/DAS. The full dataset creation plan and underlying analytic code are available from the authors upon request, understanding that the computer programs may rely upon coding templates or macros that are unique to ICES and are therefore either inaccessible or may require modification.
